# Evaluating the Reproducibility of Motion Analysis Scanning of the Spine during Walking

**DOI:** 10.1155/2014/721829

**Published:** 2014-07-17

**Authors:** Aaron Gipsman, Lisa Rauschert, Michael Daneshvar, Patrick Knott

**Affiliations:** Chicago Medical School, Rosalind Franklin University of Medicine and Science, 3333 Green Bay Road, North Chicago, IL 60064, USA

## Abstract

The Formetric 4D dynamic system (Diers International GmbH, Schlangenbad, Germany) is a rasterstereography based imaging system designed to evaluate spinal deformity, providing radiation-free imaging of the position, rotation, and shape of the spine during the gait cycle. *Purpose.* This study was designed to evaluate whether repeated measurements with the Formetric 4D dynamic system would be reproducible with a standard deviation of less than +/− 3 degrees. This study looked at real-time segmental motion, measuring kyphosis, lordosis, trunk length, pelvic, and T4 and L1 vertebral body rotation. *Methods.* Twenty healthy volunteers each underwent 3 consecutive scans. Measurements for kyphosis, lordosis, trunk length, and rotations of T4, L1, and the pelvis were recorded for each trial. *Results.* The average standard deviations of same-day repeat measurements were within +/− 3 degrees with a range of 0.51 degrees to 2.3 degrees. *Conclusions.* The surface topography system calculated reproducible measurements with error ranges comparable to the current gold standard in dynamic spinal motion analysis. Therefore, this technique should be considered of high clinical value for reliably evaluating segmental motion and spinal curvatures and should further be evaluated in the setting of adolescent idiopathic scoliosis.

## 1. Introduction

Adolescent idiopathic scoliosis (AIS) is a common condition affecting between 2 and 4 percent, or an estimated 6 million adolescents, in the United States [1]. Frequent assessment and monitoring of this patient population are necessary to determine an individual's progression of spinal deformity. Healthcare providers most often use spinal radiographs as the standard-of-care for evaluation. X-rays currently offer the most reliable way to quantify the magnitude of the curve but have the disadvantages of exposing patients to harmful radiation. Nash et al. reported that over a three-year period, a group of teenage girls with AIS underwent an average of 22 radiographs [2]. Ronckers et al. found cancer mortality to be 8 percent higher than expected in patients with repeated radiographs for scoliosis, as well as a four times greater relative risk of breast cancer in female patients with spinal disorders [3].

Surface topography is the study of the three-dimensional shape of the surface of the back. Measurement systems using surface topography do not involve exposure to ionizing radiation and are therefore completely safe [4]. According to a study by Knott et al., if surface topography can deliver reliable results, then it should replace radiographs in a certain number of follow-up clinical visits when curve surveillance is necessary and exposure to radiation can be avoided [5]. Other than the Formetric 4D machines, many surface topographical devices have been developed and tested for the purpose of screening for scoliosis [6–20].

The development of the Formetric 3D/4D device by DIERS biomedical technologies has provided a new option for static imaging of the spine. This radiation-free technology uses surface topography of the trunk to analyze surface asymmetry and identify bony landmarks thereby aiding in the evaluation of spinal deformities. As with other surface topography systems, it projects parallel stripes of light onto the back of a standing patient. The distortion of the raster lines provides the basis for calculating the surface topography. A map of 10,000 individual points is obtained and a surface is applied to these points. A large database of CT scans was used to create a mathematical model linking surface topography to spine position. The computer software contains this mathematical model and uses it to predict spinal position whenever exposed to a new topography scan. The Formetric system uses this complex algorithm to produce a three-dimensional computerized representation of the patient's spine [21]. Previous studies have indicated that patient evaluation using the Formetric 3D/4D for static measurements of spinal curvature is comparable to radiographs in terms of its test-retest reproducibility and seems to be a reliable way to monitor AIS patients [21]. According to a study performed using the static Formetric 4D machine, the trunk measurements were extremely reliable, with standard deviations consistent with those of standing radiographs. Measurements were reproducible, with standard deviations of only a few degrees for angular measurements and only a few millimeters for distance measurements [5].

Up until now, the technique of surface topography had only been applied to static imaging. However, physicians often need to analyze patients' movements to diagnose pathological or abnormal changes. Static images may underestimate the magnitude of scoliosis or general spinal curvature deformity. It would be valuable to be able to three-dimensionally analyze the spine under dynamic conditions to better understand the spinal motion in deformities such as scoliosis. Gait analysis is an option for measuring dynamic changes in spinal curvature and position; however it requires a large laboratory with expensive equipment and relies on the eye of the observer.

An alternative to gait analysis is the new version of the Formetric 3D/4D, the Formetric 4D dynamic model. Also developed by DIERS, this device uses similar surface topography techniques as the previous model to enable radiation and contact free analysis of the spine under dynamic conditions. The Formetric 4D dynamic captures images of the patient's back at a rate of 50 frames per second during simple motion (such as walking on a treadmill) for a duration of 5 seconds. Therefore, approximately 250 static images are collected and quickly converted into three-dimensional representations of the patient's spine. The combination of images results in a real-time three-dimensional representation of the shape of the spine in motion during the gait cycle. Objective values from the spine, pelvis, and scapula can be calculated, which may provide benefits in diagnosis and monitoring AIS patients and patients with deficits in postural control.

The goal of this study was to measure the reproducibility of the Formetric 4D dynamic system via analyzing the trunk length, kyphotic angle, lordotic angle, the rotation of the T4 vertebra relative to the pelvis, the rotation of the L1 vertebra relative to the pelvis, and the rotation of the pelvis during the normal gait cycle. Reproducible measurements would support the reliability of this imaging technique, which is the first step towards determining whether it should be further utilized as an effective method for screening, monitoring, and treating scoliosis, as well as other conditions affecting posture. Repeat measurements in subjects with a normal spinal curvature should be the first step in evaluating the technique's accuracy.

Measurements were considered reproducible if the average standard deviations were within +/− 3 degrees, which is the error seen with segmental orientation tracking with the VICON optical motion measurement system, the current gold standard in spinal motion analysis [22]. The VICON system uses cameras to capture real-time complex human motion by recording marker position throughout the range of motion [22]. This optical tracking system is mainly restricted to the gait laboratory setting due to the high cost and volume of equipment, but it is currently the most prevalently used method for dynamic human motion analysis [22, 23]. Thus, it is the best comparison for the reliability of this novel technique of dynamic surface topography measurement.

## 2. Significance

The justification for this research is, if an accurate nonradiographic method for measuring spinal curvature and motion can be found, clinicians may be able to reduce or eliminate the need to expose young patients to ionizing radiation during their treatment for AIS. This machine may also eliminate the need for a more formal gait laboratory analysis. Gait analysis can be expensive, time consuming, and physically tolling on patients. The goal of this study was to determine whether dynamic surface topography motion analysis can produce reliable measurements. Testing the reproducibility of this system using subjects without any known spinal deformity should be the first step in evaluating the utility of dynamic surface topography as an effective tool for clinical spinal motion analysis.

## 3. Materials and Methods

The newest version of the Formetric 4D (dynamic feature) was acquired by the Illinois Bone and Joint Institute in 2011 and the principal investigator learned to use the machine according to the manufacturer's basic recommendations. After obtaining approval from the institutional review board, all volunteers were verbally recruited from the Illinois Bone and Joint Institute and Rosalind Franklin University of Medicine and Science by the principal investigator. The main prerequisite was the ability to walk on a treadmill. Inclusion was not based on sex, race, religion, insurance, or socioeconomic status. Participants who did not understand the informed consent or did not wish to participate in the study were excluded. The study included 20 healthy volunteers (7 male and 13 female) between the ages of 20 and 27.

Initially, a pilot study was conducted in order for the investigators to learn how to use the machine better and to identify any potential problems with scanning subjects and obtaining measurements. Based on observations from this pilot study and the general protocol described by the manufacturers, a standardized procedure was developed to minimize any variability introduced by the operator. The protocol is described below.

### 3.1. Procedure

Subjects were provided with standard disposable exam shorts with elastic waistbands. The investigators ensured that shorts were positioned low enough on the hips so that the upper gluteal cleft was visible. Female subjects were provided with adhesive paper drapes to wear over their breasts. Drapes were adjusted so that they were not visible from the back. Volunteers with long hair were provided with hair clips and the investigators made sure that hair was positioned securely out of the way and completely off of the neck. All necklaces, watches, and so forth, which might have been visible in the frame of view, were removed.

#### 3.1.1. Marker Placement

**Figure 1:**
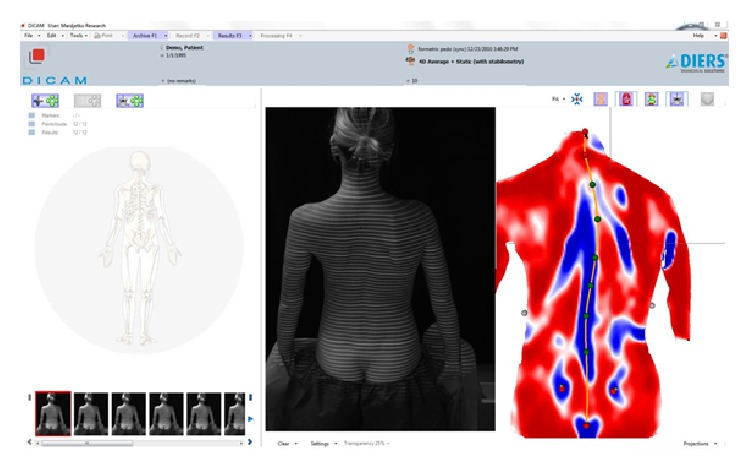
Static mode mean curvature screen: the middle image is raster line projection; the far right image is a representation of spinal curvature based surface topography.

**Figure 2:**
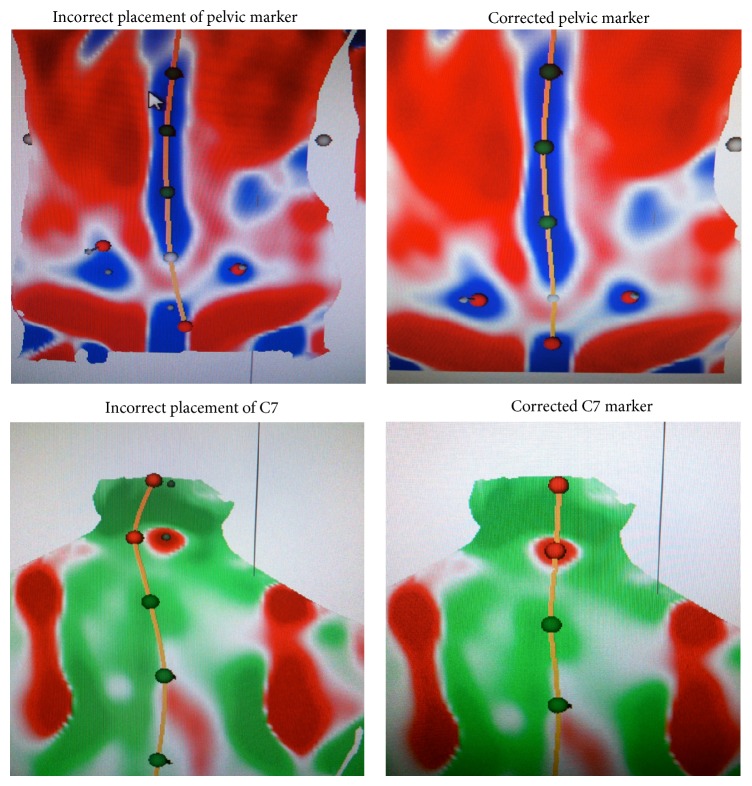
Adjustment of markers.

The primary examiner placed 3 reflective stickers on the subject's back, one on the spinous process of C7 and one on each of the posterior superior iliac spines (PSIS) in the sacral region. These markers help the machine find these points quickly allowing faster and more accurate data processing. The examiner palpated each location before placing the sticker. Proper marker placement was confirmed with static imaging as follows. After the stickers were placed, subjects were instructed to stand at a marked position on the floor in their normal relaxed position with arms at their side and back to the Formetric 4D camera. With the machine in static mode, the mean curvature screen was selected (Figure 1). The investigators confirmed that the stickers were correctly located in the center of the PSIS depressions (blue dimples) and C7 (red protuberance). The primary examiner adjusted the stickers as necessary according to the surface topography images as shown in Figure 2. Results from a previous study comparing automatic detection of anatomic landmarks using the Formetric 4D versus manual detection by the clinician showed that automatic detection using surface topography was more reliable [24]. Once the reflective stickers were properly placed, they remained there until all measurements were completed.

Next, the subject was directed to the treadmill and stood even with the tape marks indicating the 2-meter mark from the Formetric stereo imager.

#### 3.1.2. Camera Positioning and Setup

The camera column was adjusted based on the subject's height so that the spine was in center view and the green crosshairs were just below the scapula. Any lights/reflective portions of the treadmill were covered and ensured to be out of camera view. Investigators were cautious to make sure subjects' hair was still out of the way, shorts were low enough for visualization of the spine and hips, and frontal drapes for female patients were still out of camera view in this position.

**Figure 3:**
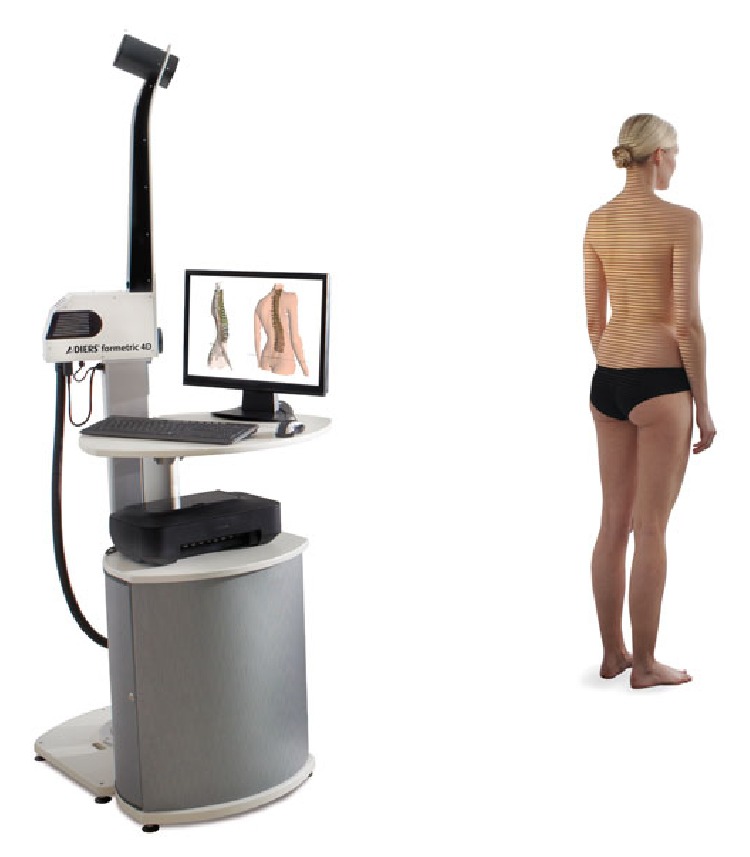
Formetric 4D device with raster line projection.

The investigator then clicked the “project stripes” button to turn on the lights. The subject's position on the treadmill was checked to make sure that the stripes of light were sharp and in focus. An example of the Formetric 4D system projecting raster lines onto a representative subject's back is shown in Figure 3.

#### 3.1.3. Lighting Conditions

Lighting in the exam room was dimmed appropriately so that raster lines projected onto the subject's back were easily visible. Best results were possible with the lights above the patient turned off and the ceiling light on the other side of the room turned on. These conditions were kept consistent for all subjects.

#### 3.1.4. Measurements

The treadmill was set to 1.8 mph and the subject began walking at a steady, comfortable pace. Investigators closely observed to make sure they were walking at the proper distance from the camera so that the stripes remained in focus and the subject was instructed to walk evenly with the 2 meter tape marks on the treadmill. After 30 seconds of walking, the examiner clicked “start recording” to begin the measurement. Once the 5-second motion image capture was complete, the lights turned off automatically. The examiner stopped the treadmill and the subject rested for 2 minutes, while the Formetric software processed the data. For each subject, these steps were repeated two more times for a total of 3 trials.

#### 3.1.5. Data

**Figure 4:**
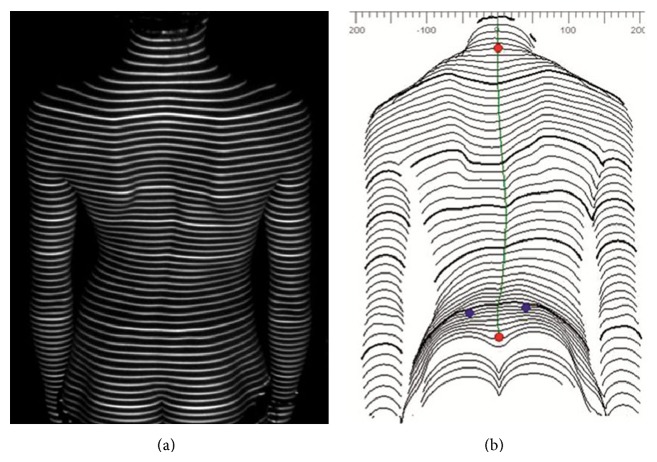
Raster lines projected onto the surface of subject's back (a) and reproduced computerized surface topography map (b).

**Figure 5:**
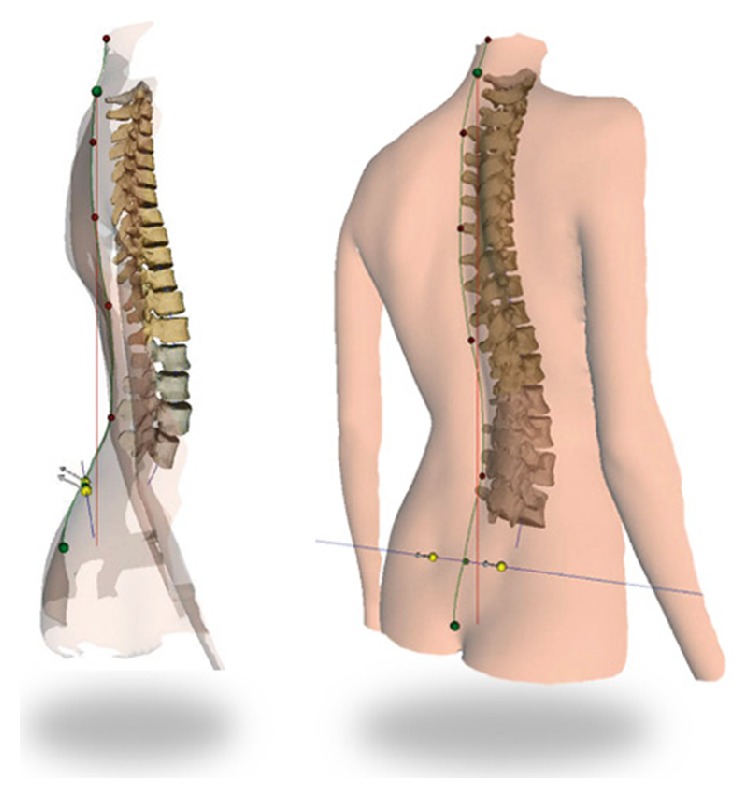
Three-dimensional computerized representation of the subject's underlying spine based on surface topography.

**Figure 6:**
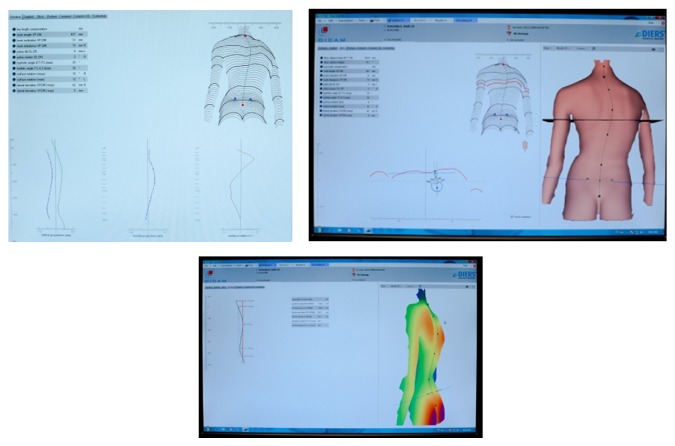
Diers Formetric sample data collection screens.

For each trial, the 5-second motion imaging capture recorded at least 3 steps of the gait cycle. Multiple measurements are recorded by the Formetric system with each trial. For this reproducibility study, the investigators looked specifically at the measurements for the maximum and minimum values of kyphosis, lordosis, the rotation of the T4 vertebra, the rotation of L1 vertebra, the rotation of the pelvis, and the trunk length. These parameters were chosen based on clinical significance and ease of obtaining measurements from the computer report. Sample screenshots showing examples of data reporting are shown in Figures 4, 5, and 6.

The investigators averaged the vertebral and pelvic rotations over the 3 steps by recording the 3 peak rotations and the 3 minimum rotations. The averages of each of these parameters from each trial were calculated. In addition, the average values of all three trials for each parameter were calculated. This was then used to calculate the standard deviation for each parameter. For each parameter, the standard deviation was taken from the three trials performed on each subject. These 20 standard deviations (one for each parameter from each subject) were then averaged to determine the average standard deviations for that parameter.

For example, the subject underwent trial number one. Two of the many parameters measured and recorded were the maximum and minimum kyphotic angle. The average kyphotic angle for trial one was then calculated and recorded using the maximum and minimum values. The subject then underwent trials two and three, and the same measurements and calculations were performed. Thus, 3 maximum, 3 minimum, and 3 average values for the kyphotic angle were measured. The 3 values of the same parameter, for example, the three maximum kyphotic angle values (one from each trial), were then averaged in order to obtain an overall average maximum kyphotic angle value for the subject. This overall average for each parameter was used to calculate the standard deviation of the three trials for that specific parameter (such as the maximum kyphotic angle). This was done for each of the parameters mentioned previously. An example of this data collection for a single subject is shown in Table 1.

## 4. Results

The average standard deviations, the standard errors of the mean, and the ranges from all 12 parameters are listed below in Table 2. When evaluating same-day repeat scans, standard deviations ranged from 0.51 to 2.3 degrees and standard error of the means from 0.14 to 0.51 degrees.

## 5. Discussion

Surface topography has been used by a number of devices for the surveillance of spinal curve progression in patients with AIS. Previous studies have already shown that the static surface topography measurement with the Formetric 4D is comparable to radiographs in estimating the position of the spine as reported by Frerich et al. [21]. Therefore this technique of dynamic surface topography, which combines multiple static images over a period of time, should be equally capable of predicting the position of the spine under the skin during a dynamic gait cycle. The advantage of the dynamic surface topography feature is that it allows for measurement of additional parameters including degree of vertebral rotations and changes in the spinal curvature throughout the gait cycle, which would be useful in certain clinical situations. This study represents the first step in determining whether dynamic surface topography scanning is a reliable tool for clinical evaluation of patients with spinal deformity.

Limitations of this study include a small sample size (*n* = 20) and the subjects being close in age, skin color, and body habitus. The standard deviation is not as relevant in small sample sizes as it is with large sample sizes. Small sample sizes also make determination of the actual mean more difficult. It is possible that out of three measurements, the middle value of the three is not the one that is most correct. However, it was the variability in the measurements that was the most relevant to the study.

Regarding the limitations of body habitus, the measurements using surface topography were shown to still be reliable when creating an accurate spinal model up to a BMI of 29 [25]. Additionally, Weiss and Seibel's article titled “*Can surface topography replace radiography in the management of patients with scoliosis?*” states “although the correlation between X-ray and surface measurements was comparable with that published before, we cannot conclude that the device can be reliably used in the surveillance of patients with AIS, as the differences in one case were as high as 38°” [26]. There is a learning curve in using the Formetric scanner, and during training of the examiner the results of the scans may not be as accurate. Additionally, there seem to be a few specific types of deformity that do not lend themselves to topographical scanning well. Extreme thoracic hypokyphosis is one of these, and for a very thin female scoliosis patient with a very flat thoracic curve, the Formetric algorithm may not be able to produce accurate results. This may have been the case with the one patient in Weiss and Seibel's study that had a 38-degree difference between the X-ray and the topographic scan.

The results showed statistically reproducible measurements for healthy subjects undergoing 3 consecutive motion scans. The measurements collected were well within +/− 3 degrees, which is also seen with segmental orientation tracking with the VICON optical motion measurement system (the current gold standard for spinal motion analysis) [23]. Therefore, the technique presented in this study should be considered clinically reproducible. The VICON system is the closest form of motion analysis tracking that is currently used in clinical practice and thus the best measure of success for this study.

## 6. Conclusion

This study was designed to evaluate the reproducibility of the technique of dynamic surface topography, in association with spinal measurements, in order to determine whether this method can be further utilized in the clinical setting. In the process of conducting the study, standardization protocols were created based on previous findings of a pilot study which evaluated variables such as lighting conditions, height and angle of camera, background materials within camera view, and positioning of clothing and long hair. The results showed a standard deviation of less than +/− 3 degrees and a range of 0.51 degrees to 2.3 degrees with SEM's less than 1 degree for all parameters studied. Thus, this technique of surface topography motion analysis was able to measure clinically relevant, reproducible, and spinal posture data during walking. It is clear that this technique provides reliable measurements for healthy subjects with same-day repeat measurements. Future studies will be aimed at determining the reproducibility of this technique among subjects with spinal deformities such as AIS, as well as evaluating the session to session reproducibility over time. This reproducibility study should potentially serve as a step in the process of the evaluation of other potential applications of this technique, such as spinal motion analysis in patients with spinal fusions, pre- and postoperatively, and the evaluation of the effectiveness of chiropractic medicine pre- and post-manipulation.

## Figures and Tables

**Table 1:** Raw data collected from one subject for all 3 trials.

	Kyphosis				Lordosis			

Trial 1	max 1	65.4	min 1	54.3	max 1	44.8	min 1	40.5
Trial 2	max 2	61.9	min 2	55	max 2	45.5	min 2	40.9
Trial 3	max 3	62.8	min 3	57.7	max 3	45.4	min 3	39.3
	Average max	**63.4**	Average min	**55.7**	Average max	**45.2**	Average min	**40.2**
	SD	1.82	SD	1.80	SD	0.38	SD	0.83

	L1 rotation				T4 rotation			

Trial 1	max 1	2.5	min 1	−0.2	max 1	5	min 1	1
	max 2	3.2	min 2	−0.4	max 2	6	min 2	0
	max 3	4.2	min 3	0.2	max 3	6	min 3	0
	Avg. max	**3.3**	Avg. min	**−0.1**	Avg. max	**5.7**	Avg. min	**0.3**
	SD	0.9	SD	0.3	SD	0.6	SD	0.6
Trial 2	max 1	3.1	min 1	0.5	max 1	7	min 1	1
	max 2	4.9	min 2	−2.4	max 2	9	min 2	−1
	max 3	3.7	min 3	−0.7	max 3	9	min 3	0
	Avg. max	**3.9**	Avg. min	**−0.9**	Avg. max	**8.3**	Avg. min	**0**
	SD	0.9	SD	1.5	SD	1.2	SD	1
Trial 3	max 1	3.8	min 1	−0.9	max 1	6	min 1	−1
	max 2	3.4	min 2	−1	max 2	7	min 2	−2
	max 3	3.4	min 3	−1	max 3	7	min 3	−2
	Avg. max	**3.5**	Avg. min	**−1.0**	Avg. max	**6.7**	Avg. min	**−1.7**
	SD	0.2	SD	0.1	SD	0.6	SD	0.6

	Pelvis rotation							

Trial 1	max 1	1.2	min 1	−2.1				
	max 2	3	min 2	−4.2				
	max 3	4.1	min 3	−3.7				
	Avg max	**2.8**	Avg min	**−3.3**				
	SD	1.5	SD	1.1				
Trial 2	max 1	3.4	min 1	−4.2				
	max 2	1.4	min 2	−5				
	max 3	2.2	min 3	−4.1				
	Avg max	**2.3**	Avg min	**−4.4**				
	SD	1.0	SD	0.5				
Trial 3	max 1	3.4	min 1	−4.5				
	max 2	2.4	min 2	−6.7				
	max 3	2.2	min 3	−3.9				
	Avg max	**2.7**	Avg min	**−5.0**				
	SD	0.6	SD	1.5				

	Trunk Length							

Trial 1	max	463.2	min	459				
Trial 2	max	463.4	min	457.3				
Trial 3	max	462.7	min	455				
	Avg max	**463.1**	Avg min	**457.1**				
	SD	0.4	SD	2.0				

**Table 2:** Average standard deviations, standard error of the mean, and range for spinal parameters studied.

Parameter	Average SD in degrees	SEM in degrees (*n* = 20)	Range of SD (in degrees)
Kyphosis maximum	2.3	0.51	0.23–6.37
Kyphosis minimum	2	0.45	0.47–6.44
Lordosis maximum	1.2	0.27	0.29–4.24
Lordosis minimum	1.2	0.27	0.1–3.07
L1 Rotation maximum	0.51	0.11	0.1–2.3
L1 Rotation minimum	0.58	0.13	0.1–2.8
T4 Rotation maximum	0.68	0.15	0–2.6
T4 Rotation minimum	0.9	0.20	0–3.6
Pelvis rotation maximum	0.62	0.14	0.1–2.7
Pelvis rotation minimum	0.63	0.14	0–3.1
Trunk length maximum	1.53	0.34	0.3–6.1
Trunk length minimum	1.3	0.29	0.4–6.0
